# Soil labile organic carbon fractions and soil enzyme activities after 10 years of continuous fertilization and wheat residue incorporation

**DOI:** 10.1038/s41598-020-68163-3

**Published:** 2020-07-09

**Authors:** Ligan Zhang, Xi Chen, Yujun Xu, Mengcan Jin, Xinxin Ye, Hongjian Gao, Wenying Chu, Jingdong Mao, Michael L. Thompson

**Affiliations:** 1 Anhui Province Key Laboratory of Farmland Conservation and Pollution Prevention, School of Resources and Environment, Anhui Agricultural University, Hefei, 230036 China; 2 Department of Chemistry and Biochemistry, Old Dominion University, Norfolk, VA 23529 USA; 3 Agronomy Department, Iowa State University, Ames, IA 50011 USA

**Keywords:** Carbon cycle, Environmental sciences

## Abstract

Labile organic carbon (LOC) fractions and related enzyme activities in soils are considered to be early and sensitive indicators of soil quality changes. We investigated the influences of fertilization and residue incorporation on LOC fractions, enzyme activities, and the carbon pool management index (CPMI) in a 10-year field experiment. The experiment was composed of three treatments: (1) no fertilization (control), (2) chemical fertilizer application alone (F), and (3) chemical fertilizer application combined with incorporation of wheat straw residues (F + R). Generally, the F + R treatment led to the highest concentrations of the LOC fractions. Compared to the control treatment, the F + R treatment markedly enhanced potential activities of cellulase (CL), β-glucosidase (BG), lignin peroxidase (LiP), and manganese peroxidase (MnP), but decreased laccase (LA) potential activity. Partial least squares regression analysis suggested that BG and MnP activities had a positive impact on the light-fraction organic carbon (LFOC), permanganate-oxidizable carbon (POXC), and dissolved organic carbon (DOC) fractions, whereas laccase activity had a negative correlation with those fractions. In addition, the F + R treatment significantly increased the CPMI compared to the F and control treatments. These results indicated that combining fertilization with crop residues stimulates production of LOC and could be a useful approach for maintaining sustainable production capacity in lime concretion black soils along the Huai River region of China.

## Introduction

Soils on Earth contain approximately 2,344 Gt organic carbon (C), which is three times more C than in the atmosphere, and soil is often regarded as the second largest organic C pool after the ocean^1–3^. The labile fractions of carbon are susceptible to short-term turnover. We identify labile organic carbon (LOC) as carbon that represents compounds that are readily decomposed by soil microorganisms^4^. There is no standard method for measuring LOC, and assessments often comprise overlapping pools of carbon that are measured as dissolved organic carbon (DOC), microbial biomass carbon (MBC), permanganate-oxidizable carbon (POXC), and light-fraction organic carbon (LFOC). Because LOC has a much shorter turnover time and greater turnover rate than more stable organic carbon in soils, it responds more quickly to changes in management practices^5^. The strong biological activity of LOC fractions means that they play a crucial role in carbon cycling, and they may be regarded as early and sensitive indicators of soil organic C (SOC) changes^5^. One method of quantifying the impacts of changes in LOC, the carbon pool management index (CPMI) of Blair et al.^6^, focuses specifically on the POXC fraction.

Crop straw residues are a source of organic carbon that influences the accumulation and decomposition of soil organic matter^7^. The quantity of crop residues has increased rapidly as the total production of cereal grains increases in the world, and it has been estimated that more than 5.3 billion tons of crop residues will be annually produced worldwide by 2020^8^. Currently, crop residues are burned in situ, incorporated into the soil, or removed from fields to be used as the raw materials for biochar, fodder, fuel, or construction materials. Incorporation of crop residues into soil is widely accepted as an effective approach to recycle and retain nutrients on a farm, helping to sustain soil fertility and prevent SOC depletion^9^. Moreover, plant residues are regarded as an important source of soil LOC and nutrients, which also have a significant influence on soil microbial activity and on the activity of extracellular enzymes^4,10^.

During decomposition of organic materials, microorganisms produce enzymes that catalyze the mineralization of SOC and convert nutrients from organic to inorganic forms^11^. Enzyme activity thus releases nutrients and influences the depletion and sequestration of SOC^12^. Previous studies^13–16^ of soils from various regions have shown that soil enzyme activities were greatly affected by C and N inputs. For example, Allison et al.^15^ found that N fertilization significantly increased the activity of cellulose-degrading enzymes, but it suppressed the activities of protein- and chitin-degrading enzymes. Jian et al.^16^ reported that N fertilization stimulated hydrolytic enzyme activities but depressed oxidative enzyme activities.

Soil enzymes (intracellular and extracellular) are widely recognized as proximate drivers of organic matter transformation and decomposition^17,18^. As key regulators of litter decomposition, soil enzyme activities may have a significant influence on soil LOC fractions^12^, and hydrolytic and oxidative enzymes may make different contributions to the formation of soil LOC fractions^19–22^ However, few studies have focused on the influence of long-term fertilization and wheat residue incorporation on the relationship between soil enzyme activities and LOC. Thus the roles of these enzymes remain unclear under field conditions.

In the present investigation, we hypothesized that long-term application of crop straw would increase soil organic matter (SOM) quality by increasing LOC fractions and by stimulating related enzyme activities secreted by microorganisms. We sought to document specific changes in LOC pools, the potential activities of soil enzymes related to straw decomposition, and the relationships among LOC fractions and enzyme activities. To address the hypothesis, we characterized the soil in a 10-year, continuous crop production experiment in a lime-concretion black soil along the Huai River where treatments consisted of chemical fertilization alone (F) as well as chemical fertilization combined with incorporation of crop straw residue (F + R). The objectives of this study were to: (1) investigate the effects of continuous F and F + R management on total SOC and labile fractions of SOC; (2) examine the effects of F and F + R treatments on potential enzyme activities related to straw residue decomposition; (3) analyze the relationships among LOC fractions and potential enzyme activities; and (4) explore the carbon pool management index (CPMI) as an early indicator of changes in SOM quality.

We measured the potential activities of the following soil enzymes: cellulase (CL), β-glucosidase (BG), acidic xylanase (ACX), lignin peroxidase (LiP), manganese peroxidase (MnP), and laccase (LA). The enzymes investigated represent important steps in the carbon cycle as the carbohydrates and lignin of plant residues are decomposed. CL, an endoglucanase, catalyzes the depolymerization of cellulose by hydrolyzing internal glycosidic linkages, while BG specifically catalyzes the release of glucose molecules from the ends of BG and similar glycans. ACX, like cellulase, is an endohydrolase that cleaves internal glycosidic bonds in xylans like those of hemicellulose. LiP, MnP, and LA are oxidative, lignin-modifying enzymes. They create small, reactive free radicals or metal cations that are capable of penetrating the phenolic lignin network, oxidizing aromatic carbon, and thus destabilizing covalent bonds to release monolignols.

## Results

### Influence of fertilizer and wheat residue incorporation on labile organic carbon fractions

The concentrations of total C, N, and labile soil organic carbon in plots of the F and F + R treatments were higher than those in the control treatment (Table 1). The total N concentrations were 43% higher in plots under the F and F + R treatments than in the control. Compared with the control, concentrations of total organic C (TOC), LFOC, POXC, MBC, and DOC in plots under the F treatment were significantly greater (P < 0.05) by 33%, 325%, 39%, 12%, and 73%, respectively, while they were significantly greater (P < 0.05) by 55%, 725%, 61%, 22%, and 154%, respectively, under the F + R treatment. Moreover, the concentrations of TOC, LFOC, POXC, MBC, and DOC in plots under the F + R treatment were 17%, 94%, 16%, 10%, and 47% higher than in plots under the F treatment (P < 0.05), respectively.

**Table 1:** Total and labile organic carbon fractions under different treatments.

Treatments	TOC (g/kg)	Total N (g/kg)	LFOC (g/kg)	POXC (g/kg)	MBC (mg/kg)	DOC (mg/kg)
Control	9.5 ± 0.2c	0.7 ± 0.02b	0.4 ± 0.1c	1.3 ± 0.1c	471.2 ± 18.5c	57.9 ± 4.5c
F	12.6 ± 0.3b	1.0 ± 0.09a	1.7 ± 0.3b	2.1 ± 0.1b	525.3 ± 8.9b	100.1 ± 12.8b
F + R	14.8 ± 0.4a	1.0 ± 0.03a	3.3 ± 0.6a	2.8 ± 0.1a	575.8 ± 9.6a	147.3 ± 9.9a

Values are shown as mean ± standard errors (n = 3).

Control: no amendment addition; F: chemical fertilization; F + R: chemical fertilization plus incorporated residues.

Different letters of a–c in a column indicate significant differences at P < 0.05.

### Influence of fertilizer and wheat residue incorporation on soil enzyme activities

Compared with the control, the F and F + R treatments increased the mean potential activities of CL, BG, LiP, and MnP, whereas these treatments decreased the potential activity of LA (Table 2). The mean activities of BG, LiP, and MnP were 64%, 47%, and 12% higher in plots under the F treatment than in the control (P < 0.05), and they were 86%, 68%, and 38% higher in plots under the F + R treatment than in the control (P < 0.05), respectively. Moreover, the activities of BG, LiP, and MnP in plots under the F + R treatment were 14%, 14% and 22% higher than those in plots under the F treatment (P < 0.05), respectively. In addition, the activity of LA was lower by 49% and 68% in F and F + R treatment plots compared to that in the control plots (P < 0.05), and the activity of LA in the F + R treatment plots was 38% lower than that in the F treatment plots (P < 0.05). The activity of ACX in the F and F + R treatment plots did not differ significantly from that in the control plots (P > 0.05).

**Table 2:** Potential activities of soil enzymes under different treatments.

Treatments	CL (μmol/d/g)	BG (μmol/d/g)	ACX (μmol/d/g)	LiP (μmol/d/g)	MnP (nmol/d/g)	LA (μmol/d/g)
Control	348.6 ± 13.3b	17.9 ± 0.4c	18.1 ± 1.1a	3.2 ± 0.2c	141.3 ± 0.4c	212.1 ± 7.8a
F	361.9 ± 3.3ab	29.3 ± 1.7b	17.5 ± 1.2a	4.8 ± 0.3b	158.3 ± 10.3b	108.6 ± 3.9b
F + R	374.2 ± 3.4a	33.3 ± 1.0a	19.0 ± 0.9a	5.5 ± 0.4a	194.2 ± 8.1a	67.2 ± 7.5c

Values are shown as mean ± standard errors (n = 3).

CL: cellulase; BG: β-glucosidase; ACX: acidic xylanase; LiP: lignin peroxidase; MnP: manganese peroxidase; LA: laccase. Control: no amendment addition; F: chemical fertilization; F + R: chemical fertilization plus incorporated residues.

Different letters of a–c in a column indicate significant differences at P < 0.05.

Potential enzyme activities per unit of MBC were used to account for differences in MBC contents, permitting a reliable comparison of soil enzyme activities under different land uses^23^. Our results indicate that the biomass-specific enzyme activities of BG in the F and F + R treatments increased by 47% and 53% relative to control treatments (Table 3). Compared with the control plots, the biomass-specific enzyme activity of CL in the F and F + R treatments decreased by 7% and 12%, respectively. The biomass-specific enzyme activity of LA in F and F + R treatments decreased by 54% and 74%, respectively.

**Table 3:** Ratio of potential enzyme activities to microbial biomass C (mean ± standard errors) under different treatments.

Treatments	CL (μmol/d/μg)	BG (μmol/d/μg)	ACX (μmol/d/μg)	LiP (μmol/d/μg)	MnP (nmol/d/μg)	LA (μmol/d /μg)
Control	0.741 ± 0.029a	0.038 ± 0.001c	0.039 ± 0.004a	0.007 ± 0.000b	0.300 ± 0.011b	0.451 ± 0.034a
F	0.688 ± 0.005b	0.056 ± 0.002b	0.033 ± 0.003ab	0.009 ± 0.001a	0.301 ± 0.015b	0.207 ± 0.004b
F + R	0.650 ± 0.016b	0.058 ± 0.001a	0.033 ± 0.001c	0.009 ± 0.001a	0.338 ± 0.019a	0.117 ± 0.012c

Values are shown as mean ± standard errors (n = 3).

CL: cellulase; BG: β-glucosidase; ACX: acidic xylanase; LiP: lignin peroxidase; MnP: manganese peroxidase; LA: laccase. Control: no amendment addition; F: chemical fertilization; F + R: chemical fertilization plus incorporated residues.

Different letters of a–c in a column indicate significant differences at P < 0.05.

### Relationships between soil organic carbon and potential enzyme activities

The correlation coefficients (*r*) between the labile organic C fractions and potential enzyme activities are given in Table 4. There were significant (P < 0.01), positive correlations between the concentrations of TOC, LFOC, DOC, MBC, and POXC and the potential activities of CL, BG, LiP, and MnP; the correlation coefficients ranged from 0.72 to 0.98. In contrast, the correlations between LA activity and the concentrations of TOC, LFOC, DOC, MBC, and POXC were negative (P < 0.01), and the correlation coefficients ranged from − 0.93 to − 0.99. There were no significant correlations between the concentrations of TOC, LFOC, DOC, MBC, or POXC and the activity of ACX.

**Table 4:** Correlation coefficients (*r*) between different labile organic carbon fractions and potential enzyme activities. For these correlations, the plots were treated as independent experimental units (n = 9).

	CL	BG	ACX	LiP	MnP	LA	TOC	LFOC	DOC	MBC	POXC
CL	1.00										
BG	0.81**	1.00									
ACX	− 0.08	0.17	1.00								
LiP	0.73*	0.94**	0.37	1.00							
MnP	0.79*	0.82**	0.46	0.86**	1.00						
LA	− 0.84**	− 0.98**	− 0.20	− 0.97**	− 0.87**	1.00					
TOC	0.83**	0.96**	0.30	0.95**	0.93**	− 0.98**	1.00				
LFOC	0.86**	0.89**	0.31	0.88**	0.98**	− 0.92**	0.97**	1.00			
DOC	0.83**	0.95**	0.32	0.91**	0.88**	− 0.93**	0.95**	0.92**	1.00		
MBC	0.87**	0.95**	0.26	0.92**	0.88**	− 0.95**	0.95**	0.92**	0.96**	1.00	
POXC	0.84**	0.96**	0.28	0.92**	0.92**	− 0.96**	0.98**	0.95**	0.96**	0.98**	1.00

TOC: total organic carbon; LFOC: light fraction organic carbon; POXC: permanganate oxidized carbon; MBC: microbial biomass carbon; DOC: dissolved organic carbon; CL: cellulase; BG: β-glucosidase; ACX: acidic xylanase; LiP: lignin peroxidase; MnP: manganese peroxidase LA: laccase.

* Correlation is significant at P < 0.05.

** Correlation is significant at P < 0.01.

**Figure 1:**
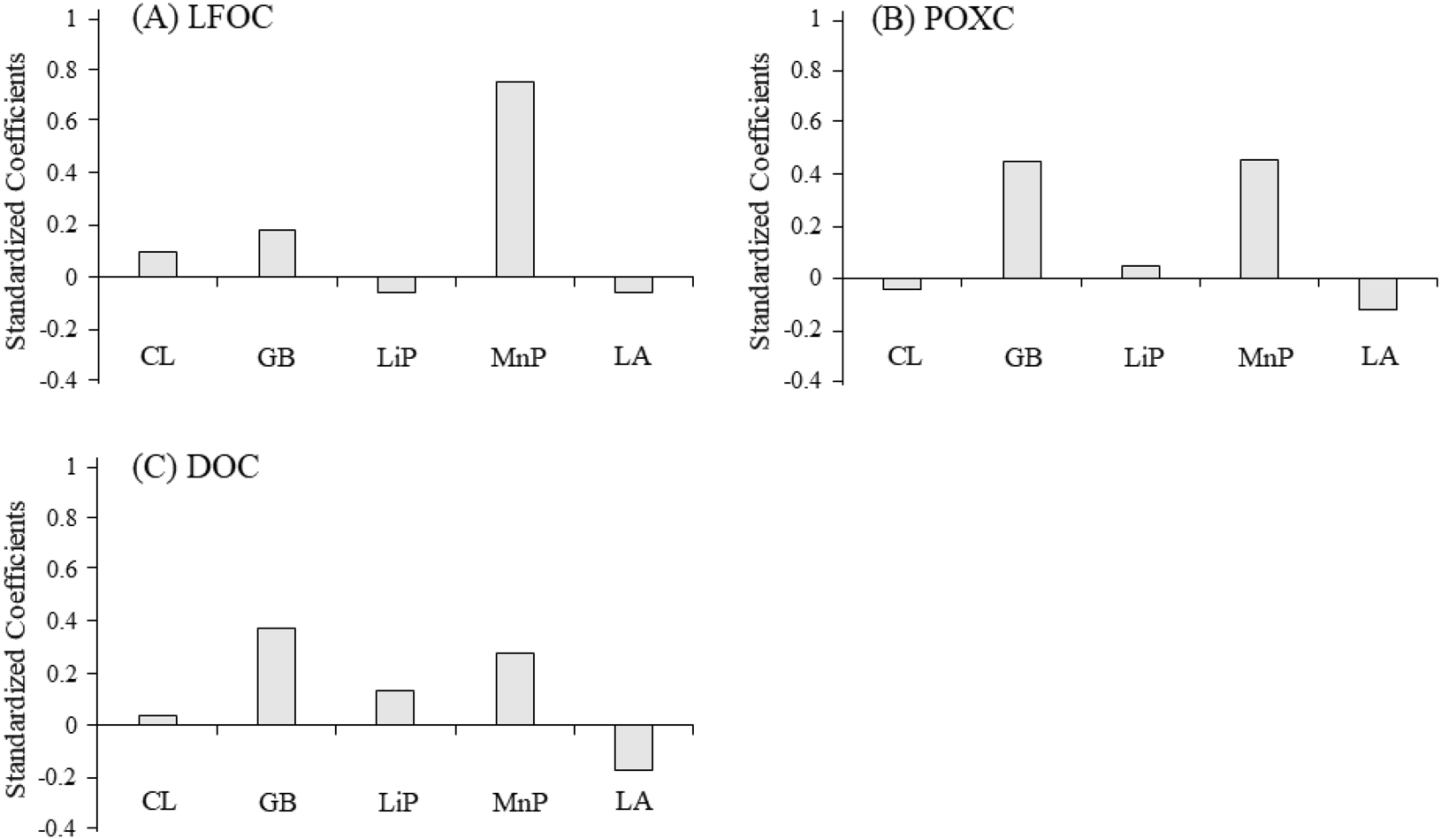
Standardized partial least squares regression coefficients showing the sign and magnitude of the impact of potential enzyme activities on concentrations of LFOC (**A**), POXC (**B**) and DOC (**C**) fractions.

Because the potential enzyme activities were correlated with one another (Table 4), partial least square (PLS) regression analysis was employed to analyze the relationships between the LOC fractions and multiple soil enzymes (Table 5). The standardized coefficients of PLS regression were used to show the sign and magnitude of enzyme activity that was related to the different LOC fractions (Fig. 1). The standardized coefficients of CL were 0.10, − 0.05, and 0.04 for the LFOC, POXC, and DOC fractions, respectively. The standardized coefficients of BG were 0.18, 0.45, and 0.38 for LFOC, POXC, and DOC fractions, respectively, and the standardized coefficients of MnP were 0.76, 0.46, and 0.28 for the LFOC, POXC, and DOC fractions, respectively. Similar to the correlation coefficients, the standardized coefficients of LA were negative, i.e., − 0.06, − 0.12, and − 0.17, for the LFOC, POXC, and DOC fractions, respectively. In general, the PLS regression analysis showed that the impacts of BG and MnP were larger than those of the other enzymes in predicting the concentrations of the LFOC, POXC, and DOC concentrations.

**Table 5:** Partial least squares regression analysis of the relationships between soil labile organic carbon fractions and soil enzymes.

Models	R^2^
LFOC = 0.010 CL + 0.035 BG − 0.080 LiP + 0.041 MnP − 0.002 LA − 9.024	0.98
POXC = − 0.003 CL + 0.044 BG + 0.029 LiP + 0.013MnP − 0.002 LA − 0.304	0.96
DOC = 0.115 CL + 2.155 BG + 5.150 LiP + 0.455 MnP − 0.152 LA − 82.127	0.96

The ACX was removed from the regression model due to its low variable importance in projection value (< 0.8).

LFOC: light fraction organic carbon; POXC: permanganate oxidized carbon; DOC: dissolved organic carbon. DOC: dissolved organic carbon; CL: cellulase; BG: β-glucosidase; LiP: lignin peroxidase; MnP: manganese peroxidase; LA: laccase.

### Influence of fertilizer and wheat residue incorporation on soil lability index, carbon pool index and carbon pool management index

**Figure 2:**
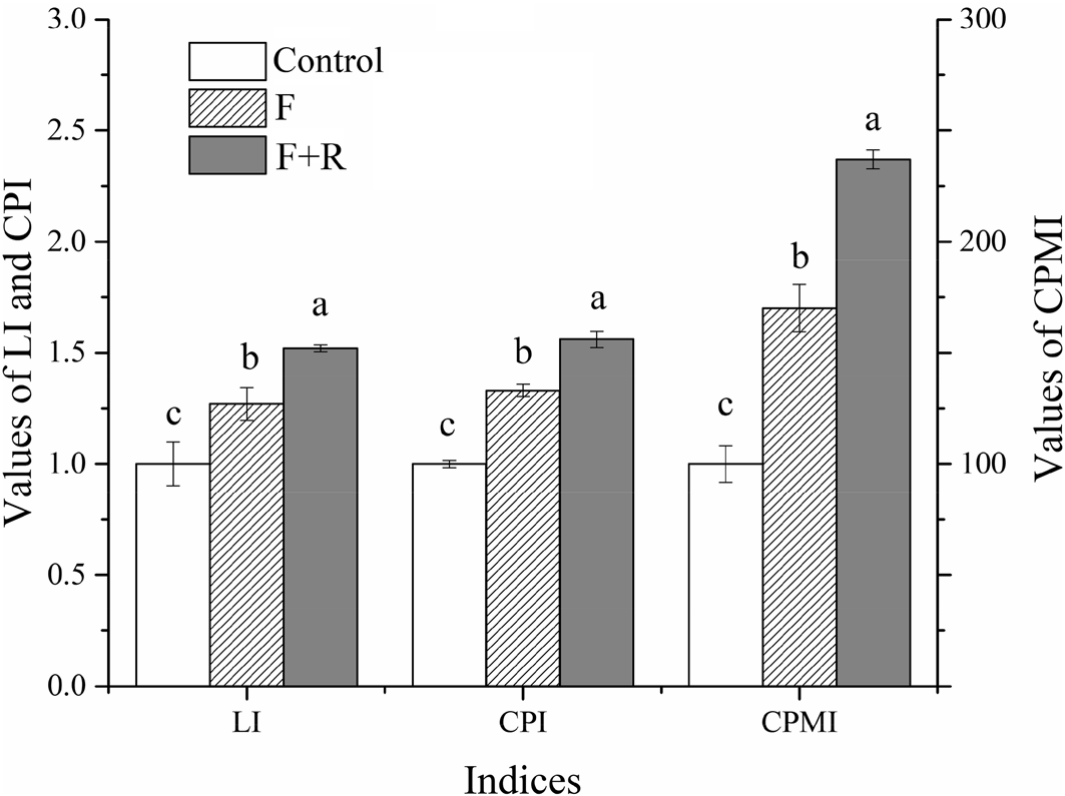
Changes in the lability index (LI), carbon pool index (CPI) and carbon pool management index (CPMI) under the different treatments. Control: no amendment addition; F: chemical fertilization; F + R: chemical fertilization plus incorporated residues. Means and standard errors are shown (n = 3). The letters a–c in a column indicate significant differences at P < 0.05.

Compared with the control plots, the values of the lability index (LI) were greater by 27% and 52% in the F and F + R treatment plots, respectively (Fig. 2). The carbon pool index (CPI) and CPMI values were greater by 33% and 70% in the F treatment plots relative to the control plots, and they were greater by 56% and 137% in the F + R treatment plots. Moreover, the CPI and CPMI in the F + R treatment plots were 17% and 39% higher than in the F treatment plots (P < 0.05).

## Discussion

### Soil labile organic carbon fractions

SOC plays an important role in improving soil fertility and sustaining soil productivity due to its effects on soil physical, chemical, and biological properties^24^. Several management practices including fertilization, return of straw, and tillage may change the quantity and quality of SOC^7,25^. Crop residues provide a source of organic matter, and when returned to soil, they often increase organic C in soil^5^. In the present study, the F and F + R treatments over 10 years of double-cropping greatly increased SOC by 33% and 56% relative to the control. This observation is consistent with previous investigations indicating that chemical fertilizer alone and crop residues or farmyard manure plus fertilizer both improved SOC levels^5,9,12^. Zhao et al.^26^ and Zhu et al.^27^ also found that crop straw return increased SOC concentrations compared to no straw return. The increase of SOC in the F treatment may be caused by an increase in belowground biomass over the non-fertilized treatment^28^. However, some studies have found the opposite results. For example, Campbell et al.^29^ reported that straw removal had no significant effect on SOC over a 10-year period. Other studies have also documented that SOC was insensitive to short-term agricultural practices because changes occurred slowly and were relatively small compared to the larger background concentrations of SOC^7,30^. Differences among these disparate studies include the initial concentration of soil C, the quantity and quality of residues and fertilizer application, single-crop or double-crop systems, the soil type and land management practices, and the period over which changes were monitored.

Overall, changes in total SOC are not easily detected in the short term because much of the bulk SOC is in forms that turn over slowly. Hence, a soil’s LOC fractions may be more sensitive indicators of changes in soil quality and functioning, because changes in their concentrations can be more easily detected in the short and medium terms^4,31^. Although they may comprise a small proportion of TOC, LOC fractions are critical components of TOC that support the biogeochemical transformation of nutrients such as N and P.

As a fraction of LOC, the LFOC is mostly derived from freshly added organic materials that are undecomposed or partially decomposed^32^; thus a high input of crop residues could increase soil LFOC. The current results show that LFOC in the F + R treatment was significantly greater than in the F and control treatments. This is consistent with the findings of Wang et al.^33^, who reported that high rates of straw return significantly increased LFOC concentrations. Incorporated crop residues are colonized by microorganisms as the residues pass through different stages of decomposition, consistent with the strong correlation between LFOC and microbial biomass (Table 4).

In the present study, the highest concentrations of DOC, MBC, and POXC were in the F + R treatment, followed by the F treatment, and the lowest in the control treatment (Table 1). This is consistent with the findings of Chen et al.^34^ and Tirol-Padre et al.^35^, who reported that high rates of straw return significantly increased the concentrations of LOC pools. Significantly higher concentrations of DOC were found in the plots where wheat straw was incorporated, which could be attributed to the release of water-soluble organic components such as hemicelluloses, monosaccharides and oligosaccharides as a result of decomposition of the straw residues^35^. Furthermore, part of the DOC could be derived from soil organic matter by the straw priming effect. The incorporation of straw or other fresh organic matter to the soil could accelerate C mineralization from pre-existing soil organic matter, thus increasing DOC^36^. Straw residue incorporation provides C-containing substrates and nutrients that stimulate the growth of microbial populations^35^.

The N fertilizer also had a significant impact on the soil labile organic carbon fractions (Table 1). Some researchers have found that both soil MBC and DOC contents decreased following inorganic nitrogen inputs. Treseder^37^, for example, conducted a meta-analysis of earlier studies and reported that the average N-induced suppression of MBC concentrations was 15%. As a result of atmospheric N deposition in temperate forests, an average reduction in DOC concentration of 22.6% was reported by Du et al.^38^. However, our results showed that fertilizer application alone increased soil LFOC, DOC, MBC and POXC content compared to the control plots. This may be because fertilization promoted the growth of both above- and belowground crop biomass and accelerated crop litter decomposition. Increased root biomass may also have led to the release of labile root exudates^39^.

### Potential enzyme activities in soil

Decomposition of plant litter and organic matter is a microbially mediated process by which microorganisms produce enzymes to catalyze the mineralization of litter residues and organic matter, converting nutrients from organic to inorganic forms^12^. Plant litter can provide energy and nutrients for soil microbial growth^36^, and as the activities of microorganisms increase, more enzymes are produced in the soil^13^. Compared to their potential activities in the control plots of this study, the potential activities of CL, BG, LiP, and MnP were greater in the F + R treatment plots (Table 2). In contrast, the potential activity of LA in the F + R treatment plots was lower than that in control plots, perhaps because N fertilization can lead to a reduction in phenol oxidase activity in soil^40,41^. Previous studies have also shown that straw return significantly increased CL and BG activities compared to a treatment with no straw return^12,25^. Our results demonstrated that the F + R treatment increased the microbial biomass without affecting the absolute ACX activities, but there were significant decreases in the potential ACX activity when normalized to microbial biomass concentration (Table 3).

Multiple enzymes catalyze the decomposition of crop residues, and each enzyme has its own substrate and ability to promote specific biochemical reactions^12^. PLS regression results revealed that BG and MnP were positively correlated with the LFOC, POXC, and DOC fractions. These enzymes, involved in decomposition of cellulose and lignin, respectively, are likely to be integral to the formation of LFOC, POXC, and DOC fractions. The larger standardized coefficient of MnP for LFOC probably reflects the vital role that MnP plays in catalyzing the breakdown of lignin in crop residues. As lignin decomposes, the hydrolase enzymes can better access other plant tissue components like cellulose, leading to an increase in LFOC, as well as POXC. In the present study, the BG activities were also positively correlated with the abundance of LFOC, POXC, and DOC. The positive correlations are also consistent with the work of Veres et al.^17^, who found positive relationships between LFOC content and soil BG activities. Similarly, Li et al.^12^ reported that cellulase and β-glucosidase activities were significantly correlated with DOC in soil (P < 0.05). In addition, the oxidase activities had a slightly positive (MnP) or negative (LA) correlation with DOC fractions. Two reasons may account for this. On the one hand, oxidase enzymes can break down lignin to form soluble phenolics, leading to direct additions to DOC^12^. On the other hand, the accumulation of phenolic compounds in the soil solution could inhibit hydrolysis reactions with dissolved organic matter, thereby leading to accumulation of low molecular weight compounds in the DOC fraction^42^. Finally, the oxidative enzymes can oxidize soluble phenolic compounds into quinones, thereby promoting humification of DOC and decreasing the soluble organic carbon in the soil solution^12,19^. Such complex and reciprocal reactions may be why MnP, LiP, and LA show different apparent relationships with the DOC and MBC fractions in the present study. While our results indicate possible relationships between enzyme activities and these LOC fractions, the specific mechanisms leading to the relationships are uncertain. Because of the small sample size, we consider these conclusions to be suggestive only and worthy of additional testing using larger datasets in comparable studies.

### The relationships among CPMI, LOC fractions and enzyme activities

The carbon pool management index (CPMI) has been used as an indicator of the response of soil organic matter to soil management changes^6^. CPMI is also an integrated measure of SOC for describing soil fertility^31^. In this study, CPMI in the F + R treatment plots significantly increased by 39% and 137% compared to the F and control treatment plots, respectively. This agrees with others’ findings that show an increase in CPMI following straw application in paddy soils^33^. Similarly, the results of Zhu et al.^27^ indicated that soils receiving straw application had higher CPMI values than those receiving mineral fertilizers alone. The CPMI increased as TOC and POXC increased in the soil, indicating the potential for a higher rate of organic matter decomposition and nutrient cycling. Straw incorporation combined with fertilization promoted BG, LiP, and MnP enzyme activities (Tables 2 and 4), increasing the soil’s POXC concentrations and therefore the CPMI values. In addition to the overall increase in LOC concentrations, the chemical composition of individual LOC pools may also change with crop straw incorporation. However, the effects of crop straw residue on the chemical composition of different LOC fractions are unknown. Further research is required to investigate compositional variations among LOC fractions under continuous incorporation of crop straw residues.

## Conclusions

Our results indicate that 10 years of continuous incorporation of wheat straw residues and application of chemical fertilizer in lime concretion black soil along the Huai River region in central China significantly increased LOC fractions, including LFOC, DOC, POXC and MBC, compared to chemical fertilizer alone or control treatments. The fertilizer plus residues treatment led to higher potential activities of CL, BG, LiP, and MnP enzymes, whereas it reduced the activities of LA. Moreover, Pearson correlation analysis showed that the concentrations of DOC, MBC, POXC, and LFOC were significantly and positively correlated with the potential activities of CL, BG, MnP, and LiP enzymes, and they were significantly and negatively correlated with potential LA activity. Partial least squares regression suggested that BG and MnP activities had large and positive partial correlations with the abundance of the LFOC, POXC, and DOC fractions, whereas LA activity had a negative partial correlation with those fractions. Furthermore, the carbon management index, CPMI, was greater under the treatment in which fertilizer was combined with wheat straw residues compared to the fertilizer only and control treatments. In summary, combining crop straw residues with chemical fertilization could be an important strategy for increasing labile fractions of soil organic matter and improving the quality of organic matter in lime concretion black soils along the Huai River region of China.

## Materials and methods

### Experimental site

A field experiment involving a double-cropping wheat-corn rotation system was conducted in Mengcheng county (33° 16′ N, 116° 55′ E) in the Huai River region in Anhui Province, China. This site has a subhumid continental monsoon climate, with a mean annual rainfall of 820 mm and a mean annual temperature of 15.5 °C. The soil is classified as a Calcic Vertisol, according to the World Reference Base for Soil Resources^43^. The basic soil properties at the beginning of the experiment in 2008 were: pH, 6.5; organic matter, 13.7 g kg^−1^; and total nitrogen, 0.76 g kg^−1^. Soil-test P and soil-test K were 22 mg kg^−1^, and 197 mg kg^−1^, respectively. The plough layer had a clay texture, with 32% sand (2 to 0.02 mm), 26% silt (0.02 to 0.002 mm), and 41% clay (< 0.002 mm).

### Experimental design

The field experiment was conducted with a winter wheat-summer maize rotation system from 2008 to 2017 using a completely randomized block design with three replicate plots per treatment. The experimental treatments at the research site included: no fertilization (control), chemical fertilizer application alone (F) and wheat straw incorporation with chemical fertilizer application (F + R). Each of the experimental plots was 49.5 m^2^.

The aboveground residues (except for stubble) from wheat and corn crops were removed completely in the control and F plots, and only corn residues were removed in the F + R plots. When the grain was harvested, the wheat straw residues (7,500 kg ha^−1^) were chopped into 5–8 cm segments by a combine-harvester machine (Qirui 4YZB-3), and then left in the F + R plots at the time of grain harvest. The crop residues were mixed with the soil by a tractor-pulled plow to the depth of 20 cm. The amount of chemical fertilizer application rates for both crops were 540 kg (N) ha^−1^ year^−1^, 157 kg (P_2_O_5_) ha^−1^ year^−1^, and 157 kg (K_2_O) ha^−1^ year^−1^.

### Soil sampling

Prior to the wheat grain harvest in 2017, five soil samples were taken from the plough layer (0–20 cm) of each plot and mixed thoroughly. Visible root and litter residues were removed from the mixed soil samples and then the mixed soil samples were air-dried and passed through a 2-mm sieve. Subsamples of fresh soil samples were stored in a cooler with freezer packs, transported to the laboratory, and stored at − 20 °C for biochemical analyses.

### Chemical analysis

Basic chemical properties of the soil samples were determined as reported by Chen et al.^25^ Soil pH was determined in 1:2.5 (w/v) soil-to-water ratio extracts. The total organic carbon (TOC) was determined by an Elementar Vario EL cube elemental analyzer through dry combustion at 900 °C and total N (TN) was determined by the Kjeldahl method. Olsen P was extracted by 0.5 M sodium bicarbonate (pH 8.5) and determined using the MoSb colorimetry method, and the exchangeable K was extracted by 1.0 M ammonium acetate (pH 7.0) and determined by flame photometry.

### Soil labile organic carbon determination

The frozen soil was thawed at room temperature for 8 h. DOC was extracted according to Jones and Willett^44^. The moist soil samples (equivalent to 5 g of oven-dried soil) (sieved < 2 mm) were shaken with distilled water at a ratio of 1:5 (weight/volume) on a reciprocating shaker for 1 h at 200 rpm. The extract was then passed through a 0.45-μm filter and stored at − 20 °C in a freezer prior to analysis. The DOC was measured using a TOC analyzer (Multi N/C 3100 TOC/TN, Analytik Jena, Germany). Before MBC analyses, the moist soils were sieved and then incubated (40% water holding capacity (WHC), 25 °C) for 10 days. MBC was determined using the chloroform fumigation–extraction method^45^. The fumigated and non-fumigated soil samples were extracted with 0.5 M potassium sulfate for 30 min. The MBC was measured by high-temperature oxidation using a TOC analyzer (Multi N/C 3100 TOC/TN, Analytik-Jena, Germany). To convert extracted C to microbial biomass C we used an extraction coefficient (K_EC_) of 0.45^46^.

POXC was measured using the 333 mM KMnO_4_ oxidation method^6^. Briefly, air-dried soil (containing ~ 15 mg of C) was added to a centrifuge tube and shaken with 20 ml of 333 mM potassium permanganate for 1 h at 200 rpm. The tube was then centrifuged for 10 min at 2000 rpm (370×*g*), and the supernatant solutions were diluted by 1:250 with distilled water. The absorbance of the diluted solutions was measured at 565 nm. The light fraction organic carbon (LFOC) was determined using the method described by Gregorich and Ellert^47^. Briefly, 10 g of air-dried soil were homogenized with 30 ml NaI solution (gravity 1.8 g cm^−3^) in a 100-ml centrifuge tube by shaking on a rotating shaker for 60 min at 200 rpm, after which it was centrifuged at 1,000 rpm (92×*g*) for 15 min. The light fraction, i.e., all floating materials after centrifugation, was transferred to a vacuum filter unit with a 20-μm nylon filter, and the material retained by the filter was washed by deionized water. This process was repeated three times. The light fractions were dried at 60 °C for organic C determinations. LFOC was determined using an Elementar Vario EL cube elemental analyzer by dry combustion at 900 °C.

### Soil enzyme activities

Before soil enzyme analyses, the frozen soils were air-dried and passed through a 40-mesh (69 µm) sieve. Cellulase (CL, EC 3.2.1.4) activity was determined by anthrone colorimetry by incubating 0.05 g soil (air-dry basis) for 3 h with carboxymethyl-cellulose solution at 37 °C^48^. β-glucosidase (BG, EC 3.2.1.21) was measured by incubating 0.02 g soil for 1 h with p-nitrophenyl-β-d-glucopyranoside at pH 6.0 (citric acid—sodium hydrogen phosphate buffer)^49^. Acid xylanase (ACX, EC 3.2.1.8) activity was determined using the 3,5-dinitrosalicylic acid (DNS) method^50,51^. The lignin peroxidase (LiP, EC1.11.1.14) activity was determined by the oxidation of veratryl alcohol^52^. Manganese peroxidase (MnP, EC 1.11.1.13) activity was determined by the oxidation of guaiacol^53,54^. Laccase activity (LA, EC 1.10.3.2) was determined by the increase in absorbance at 420 nm of 2,2′-azino-bis(3-ethylbenzothiazoline-6-sulphonic acid) (ABTS)^55^. Details of these methods are described in the supplementary information.

### Carbon pool management index

In this experiment, the carbon pool management index (CPMI) was used to monitor differences in soil C dynamics among different treatments. We calculated the CPMI and related indices following the method of Blair et al.^6^, in which the POXC fraction is considered labile C, and the non-labile C is calculated as TOC minus POXC. POXC lability (L) was then calculated as the ratio of POXC to non-labile C (Eq. 1). The lability index (LI) was computed as the ratio of the lability of an experimental treatment to the lability of the control treatment (control) (Eq. 2). The carbon pool index (CPI) compares the TOC (mg g^−1^) of an experimental treatment (C_T_) to the TOC (mg g^−1^) of the control (C_C_) (Eq. 3). The CPMI (Eq. 4) was calculated on the basis of the LI and the CPI^6^. High values of the carbon pool management index represent high amounts of labile components in the treatments compared to the control samples^6^.

1 $$ {\text{POXC lability}} \left( {\text{L}} \right) = {\text{C in fraction oxidized by KMnO}}_{{4}}/\left({{\text{Non-labile C}}} \right) $$

2 $$ {\text{Lability}}\;{\text{index }}\left( {{\text{LI}}} \right) = {\text{ POXC}}\;{\text{lability}}\;{\text{in}}\;{\text{treatment}}\;{\text{sample/POXC}}\;{\text{lability}}\;{\text{in}}\;{\text{the}}\;{\text{control}}\;{\text{sample}} $$

3 $$ {\text{Carbon}}\;{\text{pool}}\;{\text{index }}\left( {{\text{CPI}}} \right) = {\text{ C}}_{{\text{T}}} /{\text{C}}_{{\text{C}}} $$

4 $$ {\text{Carbon}}\;{\text{pool}}\;{\text{management}}\;{\text{index }}\left( {{\text{CPMI}}} \right) \, = {\text{ CPI}} \times {\text{LI}} \times {1}00 $$

## Statistical analyses

All statistical analyses were performed using SPSS version 19. Duncan’s multiple range test was used to determine which treatments were significantly different from the others. Statistical significance was assigned at the P < 0.05 level (error probability) for all statistical tests. The relationships of SOC concentrations and enzyme activities were examined by Pearson correlation analysis. The relationship between LOC fractions and multiple enzyme activities was analyzed by partial least square (PLS) regression.

## Supplementary information

Supplementary Information.
