# Supplementary material for: Soil labile organic carbon fractions and soil enzyme activities after 10 years of continuous fertilization and wheat residue incorporation
Source: Sci Rep. 2020 Jul 9;10:11318. doi: 10.1038/s41598-020-68163-3 (PMC7347534; doi:10.1038/s41598-020-68163-3)
Supplement: Supplementary file 1 — Supplementary Information. [file 41598_2020_68163_MOESM1_ESM.pdf]

Soil Labile Organic Carbon Fractions and Soil Enzyme Activities After 10 Years of Continuous Fertilization and Wheat Residue Incorporation

Ligan Zhang[1]*, Xi Chen[1], Yujun Xu[1], Mengcan Jin[1], Xinxin Ye[1], Hongjian Gao[1], Wenying Chu[2], Jingdong Mao[2], Michael L. Thompson[3]

[1]Anhui Province Key Laboratory of Farmland Conservation and Pollution Prevention, School of Resources and Environment, Anhui Agricultural University, Hefei 230036, China

[2]Department of Chemistry and Biochemistry, Old Dominion University, Norfolk, VA 23529, USA

[3]Agronomy Department, Iowa State University, Ames, Iowa 50011, USA

*Corresponding author: Ligan Zhang.

Tel: +86-551-65785852;

Fax: +86-551-65786316;

E-mail: zhligan@ahau.edu.cn

Detailed Account of Soil Enzyme Activity Analyses

Cellulase (CL, EC 3.2.1.4) activity was determined according to anthrone colorimetry.[1] Triplicate samples of 0.05 g of air-dried soil were mixed with 50 μL of toluene and shaken for 15 min. To the mixed sample, 90 μL of carboxymethyl-cellulose solution, 370 μL of Na acetate buffer and 90 μL of distilled water were added. To controls (three replicates), 90 μL of distilled water were added instead of carboxymethyl-cellulose solution. The controls and samples were shaken at 800 rpm by a rotation shaker at 37°C for 3 h, then incubated in a boiling water bath at 90°C for 15 min. After incubation, the controls and samples were centrifuged at 8000 *g* (25°C) for 10 min. After centrifugation, a portion (140 μL) of the supernatant was mixed with 260 μL of anthrone solution and incubated in a boiling water bath at 90°C for 10 min. An aliquot of 200 μL of mixed solution was transferred to a 96-hole plate, and the absorbance was determined at 620 nm. The standard curve was calculated from the glucose standard solution [1] (X: glucose concentration (mg/mL); Y: absorbance). And then the activity of CL was obtained by Equation [2] (ΔA: the difference of absorbance value between sample and control; V: reaction volume (0.6 ml); W: sample weight (0.05g); T: reaction time: 1/8 day).

$$Y = 2.5090X - 0.0462 \qquad [1];$$

$$\text{CL activity } (\mu mol/d/g) = [(\Delta A+0.0462) \div 2.5090] \times V \div W \div T \div 180.16 \times 10^{3} \qquad [2].$$

The β-glucosidase (β-GC, EC 3.2.1.21) activity was determined using *p*-nitrophenyl-β-D-glucopyranoside and measuring the absorbance of the *p*-nitrophenol.[2] Triplicate samples of 0.02 g of air-dried soil were mixed with 10 μL of toluene and shaken at 800 rpm by a rotation shaker for 15 min at room temperature (90°C for controls). To the mixed samples, 130 μL of *p*-nitrophenyl-β-glucopyranoside solution and 160 μL of citric acid - sodium hydrogen phosphate buffer solution (pH=6.0) were added. The controls (three replicates) were same to the treatment groups, except for 130 μL of distilled water were added

instead of *p*- nitrophenyl-β-glucopyranoside solution. The controls and samples were shaken at 800 rpm by a rotation shaker at 37°C for 1 h, then incubated in a boiling water bath at 90°C for 5 min. After incubation, the controls and samples were centrifuged at 10,000 *g* (25°C) for 10 min. A portion (70 μL) of the supernatant was mixed with 130 μL of Na_2CO_3 solution and transferred to a 96-hole plate, and the absorbance was determined at 400 nm. The standard curve was calculated from the *p*-nitrophenol standard solution [3] (X: *p*-nitrophenol concentration (μmol/L); Y: absorbance). The activity of β-GC by using Equation [4] (ΔA: the difference of absorbance value between sample and control; V: reaction volume (3×10^{-4} L); W: sample weight (0.02 g); T: reaction time (1/24 d)).

$$Y = 0.0016X - 0.0027 \qquad [3];$$

$$\beta\text{-GC activity } (\mu mol/d/g) = [(\Delta A+0.0027)\div0.0016]\times V\div W\div T \qquad [4].$$

Acid xylanase (ACX, EC 3.2.1.8) activity was determined using the 3,5-dinitrosalicylic acid (DNS) method.[3,4] Triplicate samples of air-dried soil (0.02 g) were combined with 100 μL of sodium acetate - acetic acid buffer solution and 50 μL of xylan mixed solution. To controls (three replicates), 50 μL of sodium acetate - acetic acid buffer solution was used instead of xylan mixed solution. The controls and samples were shaken at 800 rpm by a rotation shaker at 50°C for 30 min, and then incubated in a boiling water bath at 90°C for 10 min. After incubation, the controls and samples were centrifuged at 8000 *g* (25°C) for 10 min. An aliquot of 100 μL of supernatant was mixed with 100 μL of 3,5-dinitrosalicylic acid mixed solution, then the mixture was incubated in a boiling water bath (90°C) for 5 min. After cooling to room temperature, the absorbance of the supernatant at 540 nm was measured. The standard curve was calculated from the glucose standard solution [5] (X: glucose concentration (mg/mL); Y: absorbance). And then the activity of ACX was obtained by Equation [6] (ΔA: the difference of absorbance value between sample and control; V: reaction volume (0.15 mL); W: sample weight (0.02 g); T: reaction time (1/48 d)).

$$Y = 1.2777X - 0.002 \qquad [5];$$

$$\text{ACX activity } (\mu mol/d/g) = [(\Delta A+0.002)\div1.2777]\times V\div W\div T\div150\times10^3 \qquad [6].$$

The lignin peroxidase (Lip, EC1.11.1.14) activity was determined by the oxidation of veratryl alcohol.[5] Triplicate samples of air-dried soil (0.04 g) were mixed with 30 µL of toluene and incubated at 25°C for 15 min. After incubation, the mixed samples were combined with 100 µL of H_2O_2 solution, 60 µL of veratryl alcohol solution and 40 µL of sodium tartrate buffer (pH=3) solution. The controls (three replicates) were same to treatment groups, except for 100 µL of distilled water were used instead of H_2O_2. The controls and samples were shaken at 800 rpm by a rotation shaker at 30°C for 3 h, then incubated in an ice bath for 5 min. Samples were centrifuged at 10,000 *g* (4°C) for 10 min, and a portion (150 µL) of the supernatant was transferred to a 96-hole plate, and the absorbance was determined at 310 nm. The activity of Lip was calculated by equation [7] (ΔA: the difference of absorbance value between sample and control; ε: the molar extinction coefficient of veratraldehyde (9300 L/mol/cm); d: optical path of cuvette (0.5 cm); V: reaction volume (0.2 mL); W: sample weight (0.04 g); T: reaction time (1/8 d)).

Lip activity (nmol/d/g)= $\Delta A / (\varepsilon \times d) \times V \div W \div T \times 10^6$ [7].

Manganese peroxidase (Mnp, EC 1.11.1.13) activity was determined by the oxidation of guaiacol.[6,7] Triplicate samples of air-dried soil (0.04 g) were combined with 30 µL of toluene and incubated at 25°C for 15 min. After incubation, 120 µL of succinic acid - sodium hydroxide buffer solution, 20 µL of $MnSO_4$ solution, 40 µL of guaiacol mixed solution and 20 µL of H_2O_2 solution were added to the sample. The controls (three replicates) were same to the treatment groups, except for the succinic acid - sodium hydroxide buffer solution was used without the $MnSO_4$ solution in control samples (three replicates). The mixed samples were shaken at 800 rpm by a rotation shaker at 30°C for 3 h and then centrifuged at 8000 *g* (4°C) for 10 min. A portion (150 µL) of the supernatant was transferred to a 96-hole plate, and the absorbance was determined at 465 nm. The activity of MnP was calculated by equation [8] (ΔA: the difference of absorbance value between sample and control; ε: the molar extinction coefficient of guaiacol (12,100 L/mol/cm); d: optical path of cuvette (0.5 cm); V:

reaction volume (0.2 mL); W: sample weight (0.04 g); T: reaction time (1/8 d)).

MnP activity (nmol/d/g)= $\Delta A / (\varepsilon \times d) \times V \div W \div T \times 10^6$ [8].

Laccase activity (LA, EC 1.10.3.2) was determined by the increase in absorbance at 420 nm of 2,2'-azino-bis (3-ethylbenzothiazoline-6-sulphonic acid) (ABTS).[8] Triplicate samples of air-dried soil (0.02 g) were combined with 250 μL of ABTS solution; only 250 μL of citric acid - sodium hydrogen phosphate buffer solution was added to the controls (three replicates). The mixed samples were shaken at 800 rpm by a rotation shaker at 37°C for 10 min and then centrifuged at 10,000 *g* (4°C) for 5 min. A portion (200 μL) of the supernatant was transferred to a 96-hole plate, and the absorbance was determined at 420 nm. The activity of Lac was calculated by equation [9] (ΔA: the difference of absorbance value between sample and control; ε: the millimolar extinction coefficient of ABTS (36 L/mmol/cm); d: optical path of cuvette (0.5 cm); V: reaction volume (0.25 mL); W: sample weight (0.02 g); T: reaction time (10 min)).

Lac activity (nmol/min/g)= $\Delta A / (\varepsilon \times d) \times V \div W \div T \times 10^3$ [9].

References

[1] Sinegani, A. A. S & Sinegani, M. S. The effects of carbonates removal on adsorption, immobilization and activity of cellulase in a calcareous soil. *Geoderma* **173,** 145–151 (2012).

[2] Dick, W. A., Thavamani, B., Conley, S., Blaisdell, R. & Sengupta, A. Prediction of β-glucosidase and β-glucosaminidase activities, soil organic C, and amino sugar N in a diverse population of soils using near infrared reflectance spectroscopy. *Soil Biol. Biochem.* **56,** 99–104 (2013).

[3] Yang, Q. *et al.* Identification of three important amino acid residues of xylanase AfxynA from Aspergillus fumigatus for enzyme activity and formation of xylobiose as the major product. *Process Biochem.* **50,** 571–581 (2015).

[4] Vepsäläinen, M., Kukkonen, S., Vestberg, M., Sirviö, H. & Niemi, R. M. Application of soil enzyme activity test kit in a field experiment. *Soil Biol. Biochem.* **33,** 1665–1672 (2001).

[5] Yadav, M., Singh, S. & Yadava, S. Purification, characterisation and coal depolymerisation activity of lignin peroxidase from Lenzitus betulina MTCC-1183. *Appl Biochem. Micro.* **48,** 583–589 (2012).

[6] Anderson, A. J., Kwon, S.-I., Carnicero, A. & Falcón, M. A. Two isolates of Fusarium proliferatum from different habitats and global locations have similar abilities to degrade lignin. *FEMS microbiol. Lett.* **249,** 149–155 (2005).

[7] Camarero, S., Sarkar, S., Ruiz-Dueñas, F. J., Martínez, M. a. J. & Martínez, Á. T. Description of a versatile peroxidase involved in the natural degradation of lignin that has both manganese peroxidase and lignin peroxidase substrate interaction sites. *J. Biol. Chem.* **274,** 10324–10330 (1999).

[8] Feng, S. *et al.* Laccase activity is proportional to the abundance of bacterial laccase-like genes in soil from subtropical arable land. *World J. Microb. Biot.* **31,** 2039–2045 (2015).
